# Grain dispersal mechanism in cereals arose from a genome duplication followed by changes in spatial expression of genes involved in pollen development

**DOI:** 10.1007/s00122-022-04029-8

**Published:** 2022-02-22

**Authors:** Arthur Cross, John B. Li, Robbie Waugh, Agnieszka A. Golicz, Mohammad Pourkheirandish

**Affiliations:** 1 Faculty of Veterinary and Agriculture, The University of Melbourne, Parkville, 3010 Australia; 2 Division of Plant Sciences, The James Hutton Institute, Invergowrie, Dundee, DD2 5DA Scotland, UK; 3 Department of Plant Breeding, IFZ Research Centre for Biosystems, Land Use and Nutrition, Justus Liebig University Gießen, Gießen, Germany

## Abstract

**Key message:**

Grain disarticulation in wild progenitor of wheat and barley evolved through a local duplication event followed by neo-functionalization resulting from changes in location of gene expression.

**Abstract:**

One of the most critical events in the process of cereal domestication was the loss of the natural mode of grain dispersal. Grain dispersal in barley is controlled by two major genes, *Btr1* and *Btr2*, which affect the thickness of cell walls around the disarticulation zone. The barley genome also encodes *Btr1-like* and *Btr2-like* genes, which have been shown to be the ancestral copies. While *Btr* and *Btr-like* genes are non-redundant, the biological function of *Btr-like* genes is unknown. We explored the potential biological role of the *Btr-like* genes by surveying their expression profile across 212 publicly available transcriptome datasets representing diverse organs, developmental stages and stress conditions. We found that *Btr1-like* and *Btr2-like* are expressed exclusively in immature anther samples throughout Prophase I of meiosis within the meiocyte. The similar and restricted expression profile of these two genes suggests they are involved in a common biological function. Further analysis revealed 141 genes co-expressed with *Btr1-like* and 122 genes co-expressed with *Btr2-like,* with 105 genes in common, supporting *Btr-like* genes involvement in a shared molecular pathway. We hypothesize that the *Btr-like* genes play a crucial role in pollen development by facilitating the formation of the callose wall around the meiocyte or in the secretion of callase by the tapetum. Our data suggest that *Btr* genes retained an ancestral function in cell wall modification and gained a new role in grain dispersal due to changes in their spatial expression becoming spike specific after gene duplication.

**Supplementary Information:**

The online version contains supplementary material available at 10.1007/s00122-022-04029-8.

## Introduction

Barley was among the earliest cereal crops domesticated by humans (Harlan and Zohary 1966)*.* The process of (artificial) selection on target traits for agriculture from the wild progenitor (*Hordeum vulgare* L. ssp. *spontaneum)* occurred in the Fertile Crescent 9000 to 12,000 years ago (Tanno and Willcox 2006). The most discernible difference between the wild and the domesticated form of barley is the loss of the natural grain dispersal mechanism in the domesticated type (Pourkheirandish et al. 2015).

Grains from wild cereals progressively break off along the spike and scatter on the ground as the plant senesces and dries. In wild barley, grain dispersal occurs due to the development of a structural weak-point at each node of the central floral shaft (rachis). The weak-point presents as constriction grooves that allow mature grains to disarticulate from the rachis when a minimal external force is applied (Ubisch 1915). The functional trait of grain dispersal in wild barley is called ‘brittle rachis’. Cultivated barley does not appear to possess these constriction grooves, preventing the separation of grain from the shaft, which results in the loss of the natural grain dispersal system allowing for easier harvest. Currently, two identified genes have been associated with the non-brittle rachis characteristics in all domesticated strains of barley, the *Non-brittle rachis 1* (*btr1*) and *Non-brittle rachis 2* (*btr2*) genes (Pourkheirandish et al. 2015).

These two genes behave in a complementary dominant manner. Plants which carry a non-functional allele of either of these genes (*btr1Btr2* or *Btr1btr2*) are referred to as the non-brittle rachis type. This type has a durable spike at full maturity. While the specific molecular functions of *Btr1* and *Btr2* are unknown, an analysis of the secondary structure of the proteins predicts that they work in tandem as a receptor–ligand pair regulating the thickness of the cell wall (Pourkheirandish et al. 2015). Genomic and experimental analysis has revealed their precise location, tightly linked as a head-to-head gene pair on chromosome 3H, located 88 kb apart in the genome of the cultivar (cv.) Morex (Pourkheirandish et al. 2015). Despite their being crucial to the development of cultivated barley, no commercial cultivars carrying both mutant alleles of the *Btr* genes have yet been reported, suggesting there is no advantage to having both genes in the mutant form (Pourkheirandish et al. 2015). However, natural recombination between *Btr1* and *Btr2* has been discovered within *agriocrithon* barley (an ancestral six-row form of barley with a brittle rachis), demonstrating the possibility of genetic exchange between these two loci (Pourkheirandish et al. 2018). The presence of both *btr1* and *btr2* mutants in natural diverse barley populations demonstrates that two independent selection events have likely been made by ancient farmers during barley domestication.

Analysis of the barley genome led to the discovery of homologues of *Btr1* and *Btr2* based on the DNA sequence similarity. These genes have been named *Btr1-like* and *Btr2-like*. The physical proximity of the *Btr* gene pair and the *Btr-like* genes on chromosome 3H and their similar orientation suggests they arose by a local block duplication. *Btr1* and *Btr1-like*, as well as *Btr2* and *Btr2-like* have similar sequence, but no functional redundancy since the loss of function of *Btr* genes is not compensated by their functional *Btr-like* homologues (Pourkheirandish et al. 2015). Preliminary studies showed *Btr* expression in the rachis primordia with very limited information about *Btr-like* gene expression profiles. A study of Einkorn wheat on the corresponding gene location revealed a highly similar gene organization in the wheat genome (Pourkheirandish et al. 2017). Phylogenetic analysis of both the *Btr* and *Btr-like* genes shows that the *Btr-like* genes are ancestral to the *Btr* genes (Zeng et al. 2020). The block duplication therefore occurred before the separation of wheat and barley more than 5 million years ago and their conservation hints at a crucial function in both wheat and barley (Li et al. 2006b; Pourkheirandish et al. 2017).

The objective of this study was to determine the possible molecular function of *Btr1-like* and *Btr2-like* in barley in order to infer the ancestral functions of *Btr1* and *Btr2*. Gene expression profiling across over two hundred RNA-Seq samples showed that the *Btr-like* gene pair is expressed specifically in immature anthers. Analysis of their co-expression partners suggested roles in cell wall modification and pollen development. Together the results indicate that the *Btr1* and *Btr2* genes retained an ancestral function in cell wall modification and gained a new role in grain dispersal due to changes in spatial gene expression post-gene duplication.

## Materials and methods

### Transcriptome sample selection

We tested the transcription pattern of *Btr1-like* and *Btr2-like* in a variety of samples to determine their spatio-temporal expression. A total of 212 barley transcriptome datasets were obtained from five bioprojects selected for this analysis. Bioprojects were selected to cover a wide range of tissues and developmental time points. The tissue types found in these bioprojects cover four general plant growth stages: germination, vegetative, reproductive (non-sexual organs) and reproductive (sexual organs).

The chosen projects, listed by their NCBI accession codes, were PRJEB14349 (16 Developmental Stages, 96 samples), PRJNA558196 (Meiosis, 18 samples), PRJNA496380 (Seed Germination, 60 samples), PRJEB12540 (Drought Stress, 28 samples) and PRJNA324116 (Heat Stress, 10 samples)(Barakate et al. 2020; Cantalapiedra et al. 2017; Liew et al. 2020; Mascher et al. 2017; Pacak et al. 2016). The 16 Developmental Stages project is the largest and the most comprehensive dataset used in this study, representing a range of tissues at different developmental stages. The Meiosis project includes reproductive male organs of anther and isolated meiocyte (pollen mother cell) transcriptomes and represents genes that are involved in reproduction. The Seed Germination project contains data of barley seeds and includes tissues involved in germination. The Drought Stress and Heat Stress projects explored the gene expression profiles of barley under varying water and temperature stresses.

### Mapping reads and gene quantification

The reference genome file, Barley Morex V2 pseudomolecules, was downloaded from The Leibniz Institute of Plant Genetics and Crop Plant Research (IPK) (Mascher 2019). The RNA-Seq reads were aligned to the reference genome using HISAT2 V2.1.0 (Kim et al. 2019). The scripts are available from https://github.com/MohammadP2020/Honours-Btr-like. To quantify gene expression, read counts were normalized to Transcripts Per Million (TPM). Gene expression was quantified by TPMCalculator Version 0.0.3 (Vera Alvarez et al. 2019) using the Barley_Morex_V2_gene_annotation_PGSB.all.gtf annotation file. The TPM values for all genes in all samples were calculated.

### Co-expression analysis

RNA-Seq data were visualized using the heatmap tool maintained by the Broad Institute, Morpheus (https://software.broadinstitute.org/morpheus/). The imported file contained all 63,658 genes in the barley reference genome (Supplementary Data 1). Due to software memory restrictions, the number of genes in the table had to be reduced. Since the expression of *Btr1-like* and *Btr2-like* were highest at pachytene–diplotene in the meiocyte (more than 110 TPM for both *Btr-like* genes), any gene with a value below one TPM in that sample was removed. This adjustment removed 43,403 genes from the matrix, with 20,255 genes remaining within the dataset. This modification was sufficient for co-expression analysis in Morpheus using the ‘Nearest Neighbours’ tool and generating a heatmap.

### Sample evaluation and quality control

Hierarchical clustering (1-Pearson’s r used as a measure of distance) was performed as a quality control test to confirm clustering of within-project replicates and samples across projects. A dendrogram was generated using the inbuilt hierarchical clustering tool in Morpheus. Sample replicates that did not cluster together were removed from the analysis. The TPM values of replicates were then averaged to view how individual samples cluster together (Supplementary Data 2). The ‘Nearest Neighbours’ function in Morpheus was used to search for other genes with similar expression patterns to *Btr1-like* and *Btr2-like* using Pearson’s correlation coefficient. Genes with a similar expression, deemed by a correlation coefficient of 0.95 or larger, were selected for further analysis.

### Functional annotation using NCBI BLAST

The coding sequences of all the genes that had 0.95 or higher correlation coefficient to the expression pattern of *Btr1-like* or *Btr2-like* were extracted from Barley_Morex_V2.cds.fa using the script named get.sample.seqs.sh in conjunction with get_seqs.py, both of which are available from https://github.com/MohammadP2020/Honours-Btr-like. The resultant fasta file containing the co-expressed genes was functionally annotated using Blast2GO Version 5.2.5 (Götz et al. 2008). BLAST was performed on the sequences using the public NCBI BLAST service. This step was executed using the blastx and the non-redundant (nr) database with the default settings. Mapping was based on two databases, Gene Ontology Association and Uniprot’s ID-Mapping on 20 June 2020. Annotation was performed using the default parameters. InterProScan (IPS) was executed via the EMBL-EBI web server. The ‘Combined Graphs’ function was used to visualize the statistical information of the analysis. This analysis produced three graphs related to gene ontology (GO); biological process, molecular function and cellular component.

### Investigating potential barley–rice orthologues and homologues

A local rice database was constructed using the ‘Make Blast Database’ function available on Blast2GO with the default settings. This rice database contains the coding sequence of the Nipponbare/japonica subspecies of *Oryza sativa*, retrieved from the Genome Portal at Joint Genome Institute’s Phytozome v13 Plant Genomics Resource (JGI) (Ouyang et al. 2007).

A BLAST search was performed against this local rice database using the fasta file of the co-expressed genes to identify potential homologous sequences. This step was executed using the blastn (-task blastn) using the e-value of 1e-3 as a threshold. For genes that received multiple hits, only the top hit was retained. Alignment score was used as a measure of sequence length similarity. The equation used to calculate the alignment score is shown below.

$$ {\text{Alignment}}\,{\text{score }} = { 1}00 \, * \, \left( {{\text{Alignment}}\,{\text{length}}/{\text{Length}}\,{\text{of}}\,{\text{barley}}\,{\text{gene}}} \right). $$

An integrative approach combining sequence similarity and expression profiling was used for further functional annotation (Golicz et al. 2018). Gene pairs with a score > 60% were retained. The resulting list of genes then had their rice locus identifier recorded and then selected for further analysis by consulting the Rice Genome Annotation Project (RGAP) Version 7, where their expression profiles are available for viewing. The genes which showed an RNA-Seq Fragments Per Kilobase of transcript per Million mapped reads (FPKM) expression value higher than zero in the pre-emergence inflorescence were investigated. The pre-emergence inflorescence in rice was considered as the closest developmental stage to the anthers and meiocytes, where the expression of *Btr-like* genes was detected previously.

The rice genes that showed FPKM value larger than zero in the pre-emergence inflorescence were searched through funRiceGenes (https://funricegenes.github.io), a comprehensive database that contains functionally characterized rice genes and their related publications (Yao et al. 2017). Multiple sequence alignments were performed using online CLUSTAL multiple sequence alignment MUSCLE Version 3.8. The software calculated percentage identify through the generation of a ‘Percent Identity Matrix’. Orthologous genes were also searched within a barley–rice genome zipper (Mayer et al. 2011).

## Results

### Transcriptome sample selection

Barley RNA-Seq datasets shown in Table 1 were downloaded from NCBI and aligned to the barley reference genome using HISAT2. Alignments were of high quality, with the average overall alignment rate across all samples being 85.98% (Supplementary Data 3). The *Btr-like* genes were of primary interest in this study. While the expression data that we analysed is from different barley cultivars including cv. Morex, cv. Golden Promise, cv. Scarlett and Spanish landrace SBCC073, we utilized the reference genome and the annotation from cv. Morex.

**Table 1:** List of the RNA-Seq libraries used in this study

NCBI BioProject	Germination	Vegetative	Reproductive (Non-sexual organs)	Reproductive (Sexual organs)
16 Developmental Stages (PRJEB14349) (Mascher et al. 2017)	Developing grain *5 days post-anthesis* *15 days post-anthesis* 4-day embryo	Epidermis *Developed (4 weeks)* *Etiolated* *Senescing* Etiolated Seedling Root *Young (from seedling* *Developed (4 weeks)* Shoot *Young (from seedlings)* *Developed (4 weeks)* Tiller	Rachis Lemma Lodicule Palea	Inflorescence *Young developing (5 mm)* *Developing (10–15 mm)*
Meiosis (PRJNA558196) (Barakate et al. 2020)				Anther (mm) *0.3–0.4, 0.5–0.9, 1.0–1.2, 1.3–1.4* Meiocyte *Pre-meiosis, Leptotene–Zygotene, Pachytene–Diplotene, Metaphase–Tetrad*
Seed Germination (PRJNA496380) (Liew et al. 2020)	Scutellum * 0 h, 8 h, 16 h, 24 h, 32 h, 40 h, 48 h* Plumule * 0 h, 8 h, 16 h, 24 h, 32 h, 40 h, 48 h* Radicle * 0 h, 8 h, 16 h, 24 h, 32 h, 40 h, 48 h*			
Drought Stress (PRJEB12540) (Cantalapiedra et al. 2017)		Leaf * Control* * Mild drought* * Severe drought*		Inflorescence * Control* * Mild drought* * Severe drought*
Heat Stress (PRJNA324116) (Pacak et al. 2016)		Root * Control (22.0 °C)* * Heat stress (35.5 °C)* Shoot * Control (22.0 °C)* * Heat stress (35.5 °C)*		

**Fig. 1:**
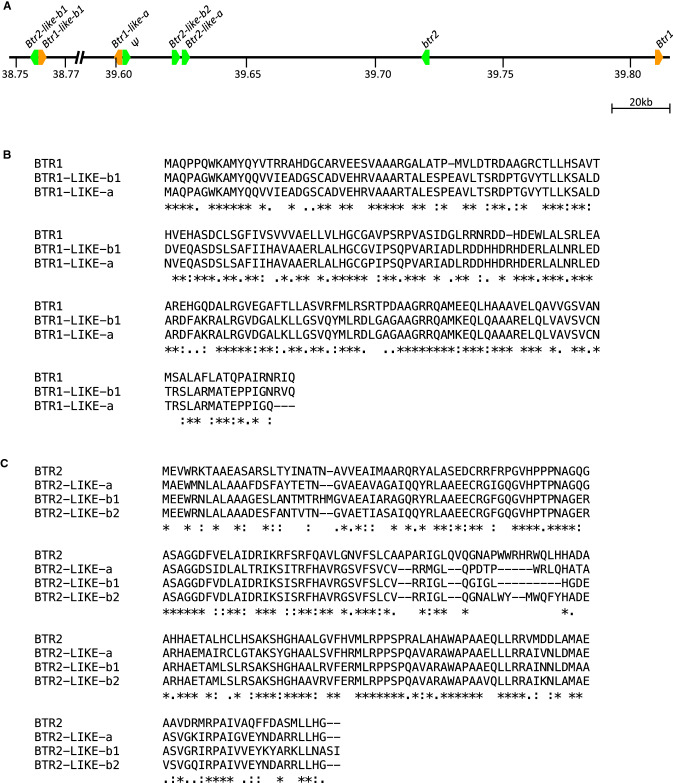
**A** The genetic map of chromosome 3H of cv. Morex (Pseudomolecules V2 annotation). Orange colour indicates sequences showing homology with the *Btr1* gene, green colour indicates sequences showing homology with the *Btr2* gene. Ψ indicates a pseudogene. **B** and **C** Amino acid sequence alignment for copies of the *Btr-like* and *Btr* genes on chromosome 3H. (**B**) BTR1: HORVU.MOREX.r2.3HG0195510, BTR1-LIKE-a: HORVU.MOREX.r2.3HG0195460, BTR1-LIKE-b1: HORVU.MOREX.r2.3HG0195170 and (**C**) BTR2: NCBI GenBank: KR813335.1 (OUH602), BTR2-LIKE-a: HORVU.MOREX.r2.3HG0195480, BTR2-LIKE-b1: HORVU.MOREX.r2.3HG0195160, BTR2-LIKE-b2: HORVU.MOREX.r2.3HG0195470 (colour figure online)

**Fig. 2:**
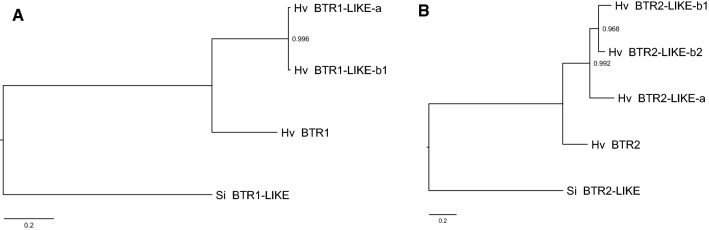
Results of evolutionary analysis of *Btr* and *Btr-like* genes. **A** Phylogenetic tree constructed using BTR1 and BTR1-LIKE protein sequences. *Setaria italica* (Foxtail millet, BTR1-LIKE (XP_004971686.1)) was used as outgroup to root the tree. **B** Phylogenetic tree constructed using BTR2 and BTR2-LIKE protein sequences. *Setaria italica* (Foxtail millet, BTR2-LIKE (XP_012701389.1)) was used as outgroup to root the tree. The evolutionary history was inferred by using the maximum likelihood method and JTT matrix-based model. Branch lengths are measured in the number of substitutions per site. Bootstrapping was performed with 500 replicates. Evolutionary analyses were conducted in MEGA X. *Setaria italica* was selected as outgroup following the multi-species phylogenetic analysis performed by Zeng et al. (2020)

The Morex annotation possessed two copies of the *Btr1-like* gene and three of the *Btr2-like* gene, all of which are potentially functional. These all exist within one Mbp of each other on chromosome 3H (Fig. 1A). *Btr1* and *Btr1-like-a* and *Btr1-like-b1*, share 58.55% and 59.18% amino acid identity (Fig. 1B). *Btr2* and *Btr2-like-a, Btr2-like-b1, Btr2-like-b2*, share 57.81%, 62.43% and 62.56% amino acid identity (Fig. 1C). According to the phylogenetic trees constructed based on amino acid alignment, *Btr1-like* gene copies are more closely related to each other than *Btr1* (Fig. 2A). Similarly, *Btr2-like* gene copies are more closely related than *Btr2* (Fig. 2B).

Gene IDs refer to the identities given in the V2 pseudomolecules annotation excluding the BTR2 because the cv. Morex does not have a functional BTR2 protein. The OUH602 BTR2 gene was used instead because its functionality has been documented (Pourkheirandish et al. 2015).

High sample quality is indicated by hierarchical clustering of similar tissue types across projects (data not shown). However, in the Drought project the Spanish landrace replicates ‘SBCC073 leaf in mild drought and heat stress’ did not cluster together and were therefore removed from further analysis. Since samples in the germination project had exceptionally high degree of similarity, the decision was made to keep only 8 h samples and the 48 h samples as representative and remove the 0 h, 16 h, 24 h, 32 h and 40 h samples. The 8 h samples were selected over the 0 h samples because they had three replicates, consistent with the number 48 h possessed. Once the above revisions were made, the TPMs values across replicates were averaged.

All *Btr-like* genes displayed similar expression patterns and their co-expression patterns are highly correlated, with above 0.95 Pearson’s correlation (Table 2). These genes show highly specific expression within the anther (zero to 145 TPM) and meiocyte (25 to 244 TPM) of barley undergoing meiosis (Table 3). No expression was detected for either of the *Btr-like* genes in any other organs used in this study (expression threshold of 1 TPM). The only exception to this specific expression pattern was low-level expression for the *Btr1-like* genes in the root samples of the Heat Stress project with maximum expression of 7.15 TPM (*Btr1-like-a* in root under 35 °C heat stress, Table 3). The expression patterns during meiosis of these copies are presented in Table 3 (expression across all samples can be found in Supplementary Data 1). The highest expression levels for all genes were present in the meiocyte at the pachytene–diplotene stage of meiosis (Table 3). We also confirmed the stage-specific expression patterns with the EoRNA database which stores expression data for 843 samples using barley gene reference transcript dataset (BaRT) and barley genome annotation V1 as a reference (Milne et al. 2021) (Supplementary Data 4).

**Table 2:** Pearson’s correlation between the expression patterns of the copies of *Btr1-like* and *Btr2-like*

Barley gene name	*Btr1-like-a*	*Btr1-like-b1*	*Btr2-like-a*	*Btr2-like-b1*	*Btr2-like-b2*
*Btr1-like-a*	1	0.99	0.99	1	0.99
*Btr1-like-b1*	0.99	1	0.97	0.99	1
*Btr2-like-a*	0.99	0.97	1	0.99	0.96
*Btr2-like-b1*	1	0.99	0.99	1	0.98
*Btr2-like-b2*	0.99	1	0.96	0.98	1

**Table 3:** Transcripts per million (TPM) values for copies of the *Btr-like* genes located on chromosome 3H in the anther and meiocyte during the early stages of meiosis and the outlier root sample from the Heat Stress project

Barley gene ID (Morex)	Gene name	Anther				Meiocyte		Root (°C)	
		Pre-meiosis	Lep–Zyg	Pach–Dip	Met–Tet	Lep–Zyg	Pach–Dip	22	35.5
3HG0195460	*Btr1-like-a*	11.87	40.75	145.34	115.23	83.01	244.05	4.58	7.15
3HG0195170	*Btr1-like-b1*	6.98	26.26	101.73	77.54	57.12	161.87	1.33	2.37
3HG0195480	*Btr2-like-a*	0	7.20	63.61	57.40	24.55	112.70	0	0.08
3HG0195160	*Btr2-like-b1*	0.95	9.19	20.41	16.30	16.61	36.04	0.96	2.98
3HG0195470	*Btr2-like-b2*	0.17	1.56	3.70	2.53	3.43	7.02	0.08	0.29

Barley Gene ID: Barley sequence name ‘HORVU.MOREX.r2.’. Lep–Zyg: Leptotene–Zygotene, Pach–Dip: Pachytene–Diplotene, Met–Tet: Metaphase–Tetrad

**Fig. 3:**
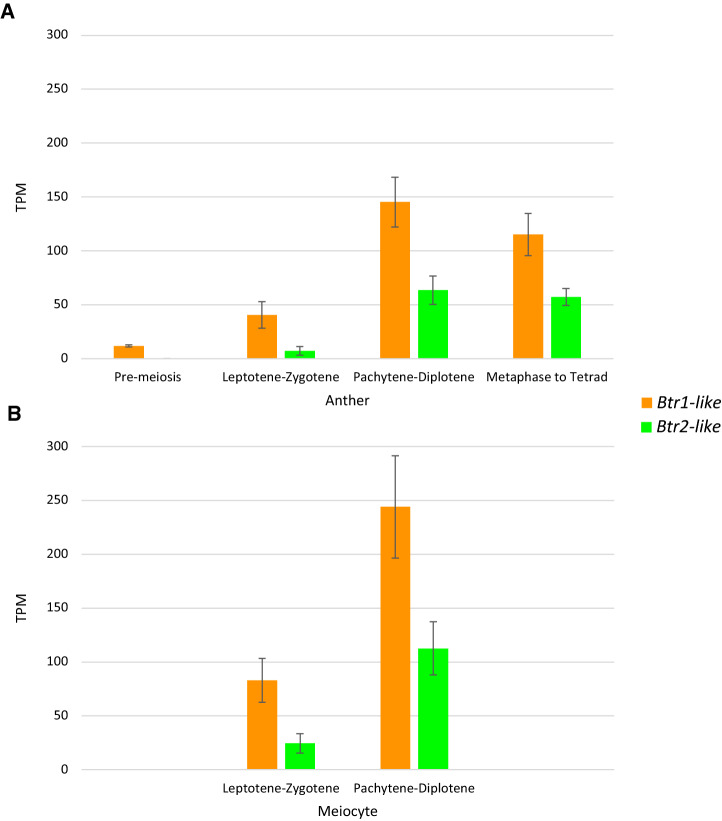
Gene expression of *Btr1-like* (orange) and *Btr2-like* (green) during meiosis of the male gametophyte (PRJNA558196) in the A: anther and B: meiocyte. Three biological samples (*n* = 3) across all stages. The TPM values for replicates were averaged to represent each sample. No expression (TPM < 1) was observed for *Btr1* and *Btr2* in the anther or meiocyte. Error bars correspond to standard error (colour figure online)

The pair with the highest expression was selected for further analysis (*Btr1-like-a* and *Btr2-like-a*)*.* Throughout the remainder of the analysis description, *Btr1-like-a* and *Btr2-like-a* are referred to as *Btr1-like* and *Btr2-like*. The corresponding expression patterns of this pair during meiosis are shown graphically in Fig. 3.

### Co-expression analysis

**Fig. 4:**
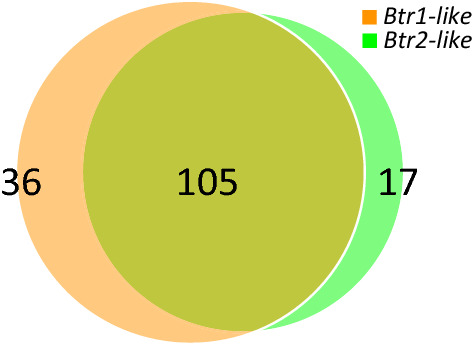
Venn diagram showing the distribution of genes co-expressed with more than 0.95 Pearson’s correlation with *Btr1-like* (orange) and *Btr2-like* (green), and the overlap between both. The gene list for each region of the Venn diagram can be found in Supplementary Data 5 (colour figure online)

We identified 141 genes correlated to the *Btr1-like* transcription pattern, and 122 genes correlated to the *Btr2-like* transcription pattern based on a positive Pearson’s correlation ≥ 0.95. Further analysis showed that 105 genes were correlated to both *Btr-like* genes (Fig. 4), suggesting that *Btr1-like* and *Btr2-like* are involved in the same molecular process. Additionally, the Pearson's correlation between *Btr1-like* and *Btr2-like* was 0.99 (Table 3).

### Functional annotation using Blast2GO

**Fig. 5:**
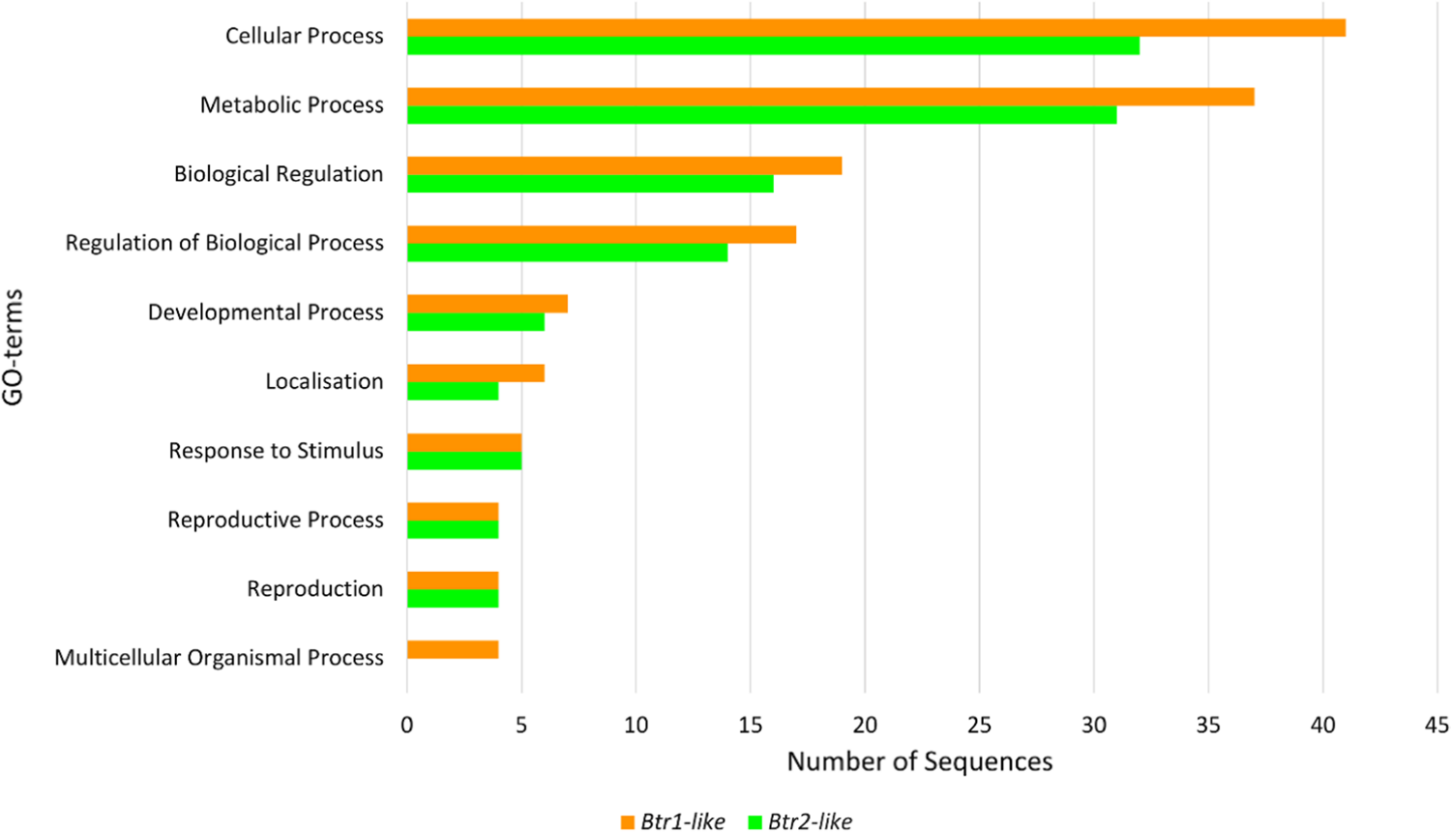
GO analysis of the ‘biological process’ category for genes co-expressed with *Btr1-like* (orange) and *Btr2-like* (green) (colour figure online)

The function of the *Btr-like* genes was inferred by analysing the molecular, cellular and biological function of genes that have similar expression patterns. The Blast2GO analysis assigned 71 genes with GO annotations for genes co-expressed with *Btr1-like* and 55 genes co-expressed with *Btr2-like*. The breakdown of the GO codes for the ‘biological process’ category is shown below (Fig. 5).

The ‘reproductive process’ category was especially interesting because of high expression levels of the *Btr-like* genes in meiocyte and anther. A total of five genes were assigned to the reproductive process GO code (GO:0022414). Four genes were annotated with GO from the correlated genes of *Btr1-like* and *Btr2-like*, three of which were co-expressed with both *Btr1-like* and *Btr2-like* (Table 4).

**Table 4:** Genes co-expressed with *Btr1-like* and *Btr2-like* that possess GO codes associated with the ‘reproductive process’ category

Barley gene ID	GO code	Co-expressed (> 0.95)
2HG0086890.1	reciprocal meiotic recombination (GO:0007131), meiosis I (GO:0007127), meiotic nuclear division (GO:0140013), meiosis I cell cycle (GO:0061982), meiotic DNA double-strand break formation (GO:0042138), meiotic cell cycle process (GO:1903046), meiotic cell cycle (GO:0051321)	*Btr1-like *and *Btr2-like*
2HG0109560.1	plant ovule development (GO:0048481), plant-type ovary development (GO:0035670), carpel development (GO:0048440), gynoecium development (GO:0048467), floral organ development (GO:0048437), floral whorl development (GO:0048438), flower development (GO:0009908), reproductive shoot system development (GO:0090567), reproductive structure development (GO:0048608), developmental process involved in reproduction (GO:0003006)	*Btr1-like *and *Btr2-like*
6HG0456990.1	anther development (GO:0048653), stamen development (GO:0048443), androecium development (GO:0048466), floral whorl (GO:0048438), floral organ development (GO: 0048437), flower development (GO:0009908), reproductive shoot system development (GO:0090567), reproductive structure development (GO:0048608), developmental process involved in reproduction (GO:0003006)	*Btr1-like *and *Btr2-like*
1HG0041780.1	seed development (GO:0048316), fruit development (GO:0010154), reproductive structure development (GO:0048608), developmental process involved in reproduction (GO:0003006)	*Btr1-like*
4HG0329610.1	anther dehiscence (GO:0009901), anther development (GO:0048653), stamen development (GO:0048443), androecium development (GO:0048466), floral whorl development (GO:0048438), floral organ development (GO: 0048437), flower development (GO:0009908), reproductive shoot system development (GO:0090567), fruit dehiscence (GO:0010047), reproductive structure development (GO:0048608), dehiscence (GO:009900), developmental process involved in reproduction (GO:0003006), multicellular organismal reproductive process (GO:0048609)	*Btr2-like*

Barley Gene ID: Barley sequence name ‘HORVU.MOREX.r2.’.

### Investigating potential barley–rice orthologues

While some of the co-expressed genes were annotated as associated with reproduction, the gene ontology analysis did not provide enough information to determine the functions for the majority of the genes (Table 4). The potential functions the 158 barley genes (Fig. 4) were investigated by comparing the gene sequences to annotated genes in the rice genome. Potential rice orthologues of barley genes were identified by selecting genes that met the following criteria (1) maximum E-value of 1e-3 in BLASTN comparisons (2) alignment score above 60%, (3) observable expression in pre-emergence inflorescence (FPKM > 0) (4) detailed functional characterization described in the funRiceGenes database. A total of 19 genes met all the criteria, 16 of which were associated with *Btr1-like*, 10 with *Btr2-like* and seven with both (Table 5). A full list of 55 genes fitting criteria (1) to (3) can be viewed in Supplementary Data 6.

**Table 5:** Function of the potential rice orthologue of co-expressed genes of both *Btr1-like* and *Btr2-like*, only *Btr1-like* and only *Btr2-like*

Barley Gene ID	Rice Gene ID (MSU)	e-Value	Rice gene	Molecular function (rice gene)	Correlation (*Btr1-like*)	Correlation (*Btr2-like*)
1HG0038070.1	10g36170.1	1.76E-48	*OsLTP2.12*	Lipid transfer protein	0.95	0.95
1HG0057890.1	05g39560.1	0	*OsZIP5*	Zinc transporter	0.96	0.97
2HG0109560.1	07g38410.1	5.16E-109	*OsYABBY7*	Summary of YABBY family genes	0.99	0.98
3HG0222470.1	01g40630.1	0	*LOG*	Maintain meristem activity	0.98	0.97
4HG0324070.1	03g15880.1	0	*OsCOI2*	Jasmonate signal transduction	0.96	0.95
4HG0325010.1	03g15340.1	6.57E-106	*UCL7*	Uclacyanin-like protein family	0.95	0.96
6HG0456990.1	02g02820.1	0	*TDR*	Tapetum degeneration retardation	0.98	0.96
1HG0041780.1	10g42550.1	0	*ITPK5*	Phytic acid biosynthesis	0.96	0.93
3HG0197830.1	07g28850.1	0	*AGO18, OsAGO18*	Male gametophyte development by small RNA-mediated mechanism, virus resistance	0.95	0.91
3HG0256970.1	01g65790.1	0	*OsPME7*	Pectin modification, root elongation inhibition	0.96	0.93
4HG0289080.1	11g08200.1	3.15E-52	*OsAsp1*	Aspartic acid regulation, Nucellin programmed cell death and meristematic development	0.96	0.94
4HG0343230.1	06g10990.1	1.61E-101	*S5(ORF3)*	Rice wide compatibility gene S5	0.96	0.92
4HG0347150.1	03g01160.2	0	*OsPUB50*	U-box-containing proteins	0.95	0.93
6HG0477800.1	09g26660.1	0	*OsrbohB, Osrboh7*	ROS production	0.95	0.94
6HG0498590.1	02g44250.1	0	*OsINP1*	Rice pollen aperture formation	0.95	0.93
7HG0586190.1	08g01410.1	0	*OsUGT2*	Sugar transporter	0.95	0.94
7HG0622890.1	10g25310	0	*OsSPX3*	Overexpression of OsSPX3 downregulates OsSPX5 in shoots under Pi-sufficiency	0.93	0.95
5HG0413550.1	02g28870.1	0	*OsPUB73*	Tapetum and exine abnormalities	0.93	0.96
2HG0132740.1	04g30470	0	*OsPUB76*	Summary of U-BOX-containing proteins	0.94	0.95

Barley Gene ID: Barley sequence name ‘HORVU.MOREX.r2.’. Rice Gene ID: Locus ID of matched rice gene ‘LOC_Os’. Key message of rice genes retrieved from https://funricegenes.github.io on 05/10/20

Among the 19 genes shown in Table 5, there were six well-described genes that are involved in pollen or reproductive tissue development in rice. These were *TDR*, *OsYABBY7, OsPUB73, OsINP1*, *ITPK5* and *OsAsp1*. Multiple sequence alignment compared the amino acid sequence between the rice genes and their prospective orthologous/homologous sequence in barley and wheat are presented in Table 6. The full sequence alignments between barley, rice and wheat can be found in Supplementary Data 7. Investigation of *OsAsp1* revealed two homologues in barley, for which a multiple sequence alignment and percentage similarity between them can be viewed in Supplementary Data 8.

**Table 6:** Amino acid sequence identity of potential rice orthologues of co-expressed genes of *Btr1-like* and *Btr2-like* with potential orthologues from wheat, *Brachypodium* and sorghum

Barley Gene ID	Rice gene name	Barley–rice amino acid identity (%)	Wheat gene accession code (NCBI)	Barley–wheat amino acid identity (%)	Brachypodium Gene ID	Sorghum Gene ID	Molecular function in rice	Biological function in rice
6HG0456990.1	*TDR*	64.88	KAF7088250.1	92.41	3g01901.1	04g001650.1	Transcription factor	Pollen development, cell death
2HG0109560.1	*OsYABBY7*	66.67	KAF7015187.1	94.51	–	–	Transcription factor	Morphogenesis, ovule development
5HG0413550.1	*OsPUB73*	47.28	KAF7060109.1	88.51	–	–	Ubiquitin ligase	Programmed cell death in anther
6HG0498590.1	*OsINP1*	85.10	KAF7079703.1	96.28	3g50820.1	04g032730.1	Unknown	Morphogenesis, pollen aperture development
1HG0041780.1	*ITPK5*	78.36	KAF6983924.1	97.41	–	01g028090	Kinase	Drought tolerance
4HG0289080.1	*OsAsp1*	48.55	KAF7052952.1	85.84	–	–	Aspartic acid protease	Programmed cell death

Barley Gene ID: Barley sequence name ‘HORVU.MOREX.r2.’. *Brachypodium* Gene ID: ‘Bradi’. Sorghum Gene ID: ‘Sb’

Sorghum and *Brachypodium* orthologues were found using synteny maps contracted using the genome zipper approach (Table 6). This analysis revealed potential orthologues of *Brachypodium* for *TDR* and *OsINP1*, while potential orthologues of sorghum were identified for *TDR*, *OsINP1* and *ITPK5* (Mayer et al. 2011).

## Discussion

### The brittle rachis is a unique mechanism evolved in Triticeae tribe via a gene duplication

Wild barley possesses a natural grain dispersal mechanism called the brittle rachis, which is governed by the presence of two tightly linked genes named *Btr1* and *Btr2* (Pourkheirandish et al. 2015). These genes are integral to the formation of the separation zone for the disarticulation of mature grain from the rachis. This separation zone presents due to the thinning of cell walls along the separation zone. The *Btr1* and *Btr2* genes are only found within some members of the Triticeae tribe including wheat and rye (Zeng et al. 2020). The presence of *Btr1* and *Btr2* correlates well with the brittle rachis characteristic (Sakuma et al. 2011). The unique separation mechanism (brittle rachis) and exclusive nature of gene sequences suggest that the *Btr1* and *Btr2* genes evolved in Triticeae which ultimately resulted in the development of a new dispersal mechanism. This hypothesis is further supported by the existence of duplicated copies of *Btr1* and *Btr2* with high sequence similarity in the barley genome known as *Btr1-like* and *Btr2-like*. Preliminary analysis has indicated that these *Btr-like* genes are not functionally redundant with *Btr1* and *Btr2.* A functional *Btr1-like* gene cannot compensate the loss of function at the *btr1* locus and similarly the loss of function of the *btr2* locus cannot be compensated by a functional *Btr2-lik*e gene (Pourkheirandish et al. 2015). The *Btr1-like* and *Btr2-like* genes are thought to be the ancestral copies, with phylogenetic analysis showing that genes sharing homology with the *Btr-like* are found throughout the Poaceae family. In contrast, the *Btr1* and *Btr2* were limited to the Triticeae tribe (Zeng et al. 2020). Here, a bioinformatics approach was adopted to uncover the potential role of the *Btr-like* genes that may then shed light on the evolution and molecular function of *Btr1* and *Btr2*.

### The *Btr*-like genes are exclusively expressed in plant reproductive organs

A comprehensive analysis of barley transcripts revealed a highly specific expression pattern of the *Btr-like* genes. They are both exclusively expressed throughout the early meiotic stages of pollen development, indicating that they perform a crucial biological role in the reproductive process in barley. Additionally, the *Btr1-like* and *Btr2-like* genes have a synchronous expression pattern that supports their involvement in related biological processes. The expression of the *Btr2* gene has been detected in cells at the rachis node during spike development at early white anther stage (Pourkheirandish et al. 2015). The immature spike in this stage is around 3–5 mm in length. The early white anther stage in spike coincides with the meiosis stage within the anthers. This suggests that *Btr* and *Btr-like* genes are expressed at a similar stage. However, the expression location differentiates the *Btr2* expressed at rachis node from the *Btr-like* genes (expressed within anthers), suggesting they have different biological roles within barley. For *Btr* genes, the outcome is rachis disarticulation, which occurs during spike maturity much later than the *Btr* loci expression. This suggests that BTR proteins were required to create a separation zone during spike development that can break at the later stage. The *Btr-like* transcripts are exclusively found in anthers, but it is yet to be determined if their biological function is immediately pronounced at the same stage as their expression, or much later akin to the *Btr* genes. The co-expression pattern of *Btr1-like* and *Btr2-like* genes supports a possible receptor–ligand pair relationship similar to that hypothesized for the BTR1 and BTR2 proteins (Pourkheirandish et al. 2015). It is tempting to speculate that the *Btr1-like* and *Btr2-like* unit was duplicated and then the copies functionally diverged to express in a different organ at a similar time (stage) with similar molecular function resulting in the *Btr* genes (Zeng et al. 2020).

Considering the above hypothesis, any information regarding the molecular function of *Btr-like* genes will be helpful to determine the molecular function of the brittle rachis. The highly specific expression patterns and homology of co-expression partners to known rice genes were used to infer biological processes of *Btr1-like* and *Btr2-like*.

### The biological function of *Btr1*-like and *Btr2*-like based on the co-expressed genes

Rice is the best model crop in monocots possessing a detailed annotated genome. It has a relatively close relationship with barley, diverging from a common ancestor 50 million years ago (Middleton et al. 2014). The biological function of the genes co-expressed with *Btr1-like* and *Btr2-like* was investigated using homologues in rice. There were six well-described genes in rice, where the barley putative orthologues were co-expressed with *Btr-like* and appeared to be involved in a similar biological process within pollen or reproductive tissue development.

Out of 158 genes co-expressed with at least one of the *Btr1-like* and *Btr2-like* in barley, 55 met our criteria for matches in the rice genome (Supplementary Data 6) and 19 had entries in the funRiceGenes database. Six were found to have well-characterized functions in reproduction, which allows us to make inferences on the overall biological function of the *Btr-like* genes. These rice genes are named *TDR* (*TAPETUM DEGENERATION RETARDATION*), *OsYABBY7* (*Oryza sativa YABBY 7*), *OsPUB73* (*Oryza sativa PLANT UBIQUITIN-BOX 73*), *OsINP1* (*Oryza sativa INAPERTURATE POLLEN 1*), *ITPK5* (*INOSITOL 1,3,4-TRISPHOSPHATE 5/6-KINASE*) and *OsAsp1* (*Oryza sativa ASPARTIC PROTEASE 1*). Here, we tried to speculate the function of *Btr-like* genes based on their molecular partners with similar expression patterns.

The TDR protein is a putative basic helix loop helix (bHLH) transcription factor (Li et al. 2006a). It is preferentially expressed in the innermost layer of the anther (the tapetum) in rice. The tapetum is in direct contact with the developing pollen. Expression of *TDR* is specific to the anther and begins early in meiosis and peaks at the young microspore stage that is similar to the barley *Btr-like* genes demonstrated in this study. Naturally, in rice, the tapetum is almost completely degenerated by the vacuolated pollen stage allowing pollen the space to develop to maturity. However, in the loss of function mutation (*tdr*) the tapetum does not degenerate as in the wild-type, swelling and destroying the meiocytes in the process. *TDR* silencing appears to be linked to the retardation of programmed cell death in the tapetum, meiocyte abortion and pollen wall abnormalities (Li et al. 2006a; Zhang et al. 2008). The most striking morphological defect is the delayed degeneration of the tapetum and results in complete male sterility. The pollen wall abnormalities have also been further investigated, revealing that these defects occurred after forming the primexine layer (micro-fibular matrix that forms the mould for the pollen wall deposition) by microspores (Blackmore and Barnes 1985; Echlin and Godwin 1968; Zhang et al. 2008). Barley has a prospective orthologue of *TDR* within the candidate gene list. Additionally, the barley orthologue was one of the genes identified in BLAST2GO analysis with the GO code for anther development. Multiple sequence alignment of the pair revealed that barley and rice *TDR* share amino acid sequence identity of 64.88%. The expression patterns of rice *TDR* and the barley orthologue temporally mirror each other. Since *TDR* is primarily expressed in the tapetum and the barley orthologue expressed in the meiocyte sample, this finding indicates that tapetal tissue was likely included in the sample (the membrane of the ‘meiocyte bag’) (Barakate et al. 2020). Rice *TDR* is located on chromosome 2, in syntenic position with the barley orthologue located on chromosome 3H (Mayer et al. 2011). Moreover, *TDR* has orthologous sequence not only in barley but *Brachypodium* and sorghum as well (Bradi3g01901.1 and Sb04g001650.1). This indicates that the gene is well conserved across monocots and, therefore likely has a conserved and crucial function as demonstrated in rice.

The OsYABBY7 protein is part of the YABBY gene family; the zinc finger transcription factor family, which has been known to play important biological roles in morphogenesis, growth and development (Zhao et al. 2020). However, real-time PCR expression analysis of the YABBY genes in rice revealed that *OsYABBY7* has a significantly lower-level but more targeted expression than the other *YABBY* genes (Toriba et al. 2007). *OsYABBY7* is specifically expressed in the reproductive organs of flowers whereas the other *OsYABBY* genes have more general expression across all inflorescence structures and other meristematic tissues. Barley has a prospective orthologue of *OsYABBY7* within the candidate gene list and the barley expression levels seen in the data strongly support similar expression patterns to rice. The barley orthologue was also identified within the BLAST2GO analysis with the GO code for plant ovule development. Multiple sequence alignment of these sequences revealed an amino acid sequence identity of 66.67%. This gene is not well studied and would be a good candidate for further research.

The rice protein OsPUB73 was first identified with the U-box domain and putative function as an E3 ubiquitin ligase (Zeng et al. 2008). This protein domain has been linked with regulating programmed cell death signalling (Zeng et al. 2008). However, the context in which *OsPUB73* has been expressed and the biological pathway it is involved in has only recently been investigated. It has shown high expression in the anther throughout the early stages of meiosis. Gene silencing of *OsPUB73* in rice revealed reduced pollen fertility, incomplete tapetum degeneration/cell death and pollen exine abnormalities (Chen et al. 2019). The best match to rice *OsPUB73* gene in barley genome is the candidate gene found in the current co-expression study and the multiple sequence alignment revealed the genes share a 47.28% amino acid sequence identity. The expression pattern of the putative barley orthologue is consistent with that of the rice gene.

While both *TDR* and *OsPUB73* are important for the development of fertile pollen neither of these genes prevents the meiotic process from occurring. The meiocytes in both mutant variants (*tdr* and *ospub73*) reach the tetrad stage without any obvious irregularities despite both genes being expressed well before the tetrad stage. While both genes cause similar abnormalities when defective, comparative co-expression analysis between mutant variants *ospub73* and *tdr* indicate that these genes could function in independent molecular pathways in rice (Chen et al. 2019). It is also possible that *OsPUB73* is part of the same functional process as *TDR* but exists further downstream and does not affect the regulation of as many genes. Based on the co-expression, one can hypothesize that *Btr-like* genes are also involved in pollen cell wall development during the meiosis stage.

The putative function of *OsINP1* is unknown, but its role in overall biological processes has been described in a rice gene knockout experiment (Zhang et al. 2020). Expression of *OsINP1* is present in meiocytes in rice from the meiocyte stage to the free microspore stage (Zhang et al. 2020). Stained confocal microscopy revealed *OsINP1* is localized to distal poles of the tetrad. Areas with high concentration mark points of depleted pollen precursor deposition, which appears to only occur at the position of the pollen aperture development. Pollen apertures are small openings on the pollen surface that allow the pollen tube to emerge from inside the pollen during fertilization (Edlund et al. 2004). It was demonstrated that while a mutation to *OsINP1* does not affect the development of anthers and pollen grains, the aperture in mature pollen grains is absent in mutants. This prevents the emergence of the pollen tube, resulting in male sterility. Barley has a prospective orthologue of *OsINP1* within the candidate gene list. The expression levels of the barley gene seen within this analysis are consistent with the function of *OsINP1* seen in rice. Multiple sequence alignment analysis of *OsINP1* and its potential barley orthologue reveal an 85.10% amino acid sequence identity. This gene is well conserved and has been identified in the barley–rice zipper genome synteny model, and *Brachypodium* and sorghum (Mayer et al. 2011).

The gene *ITPK5* is a part of the ITPK gene family first uncovered in *A. thaliana.* This gene family appears to be exceptionally well conserved across monocot and dicot species. The ITPK gene family encodes a putative inositol 1,3,4-trisphosphate 5/6-kinase which is involved in polyphosphate biosynthesis and is part of the broader ATP-grasp proteins (Fawaz et al. 2011). Detailed studies in rice have been undertaken on the homologue *ITPK2,* which has been linked to response to drought tolerance. An expression comparison between *ITPK* genes present within rice (*ITPK1-6*) indicated that *ITPK5* showed the highest expression within the endosperm, 7 and 14 days after pollination (Du et al. 2011). The expression analysis of the barley orthologue also shows expression in the developing grain (consistent with the endosperm), much like the rice orthologue. However, the barley orthologue exhibits an increased expression level during the early stages of meiosis that is not observed in the rice orthologue. GO codes associated with potential barley orthologue *ITPK5* indicate it is involved in seed development. The difference in expression from the rice *ITPK5* to the barley orthologue suggests that it may have additional biological function in barley. Multiple sequence alignment analysis of *ITPK5* and its barley counterpart reveal a 78.36% amino acid sequence identity and it is located within the synteny model for sorghum (Mayer et al. 2011).

*OsAsp1* appears to be a homologue of a barley gene within the candidate gene list. This gene’s molecular function is to produce an aspartic acid protease (Bi et al. 2005). This is a proteolytic enzyme that breaks down proteins that function optimally in acidic environments (Cao et al. 2019). It is known to be associated with meristematic activity and has been linked to various reproductive tissues: nucellus, embryos, ovary walls and the coleoptiles of immature seeds. Previous studies have indicated that this gene was more specifically involved in programmed cell death, however this has been disputed (Bi et al. 2005; Chen and Foolad 1997). There appear to be two homologues of *OsAsp1* in barley, one is the barley ‘nucellin’ gene, the other is the gene on our candidate list that we will term nucellin-like. Multiple sequence alignment analysis of *OsAsp1*, nucellin and nucellin-like proteins reveals a 57.84% identity between *OsAsp1* and nucellin, and a 48.27% identity between *OsAsp1* and nucellin-like. This indicates that nucellin is the likely orthologue of *OsAsp1* not the nucellin-like that is co-expressed with *Btr-like* genes*.* While sharing homology, nucellin and nucellin-like are also only 48.97% similar, suggesting that nucellin-like may exhibit a diverged function compared to nucellin.

### Potential biological processes involving BTR-LIKE proteins

The potential biological pathway of the *Btr-like* gene pair can be elucidated based on the strong correlation of expression between *Btr-like* genes and the three barley orthologues of *TDR, OsPUB73* and *OsINP1*. These three genes are all attributed to pollen and pollen wall development. The other three genes, *OsYABBY7*, *OsAsp1* and *ITPK5*, do not have sufficient information to analyse further and would require further research.

The tapetum directly surrounds the developing pollen. However, throughout meiosis, a callose wall is constructed between the meiocytes and the tapetum, preventing nutrient flow (Fernández 2012). In the late tetrad stage, the tapetum begins releasing callase enzymes to break down the callose wall and starts excreting precursors to the pollen wall (Ünal et al. 2013). Callose and cellulose are polysaccharides that are constituent components of the plant cell walls, although callose is less abundant than cellulose (Ünal et al. 2013). The timing of callose and callase secretion has been tied to male sterility in *Petunia hybrida* and sorghum, indicating that tight regulation of these processes is imperative to the successful creation of fertile pollen (Ünal et al. 2013). A hypothesis exists that the callose wall also acts as a template for the primexine layer in the developing pollen (Ünal et al. 2013; Waterkeyn and Bienfait 1970). This suggests that any disruption with this biological process will likely result in pollen coat abnormalities.

While barley and wheat are closely synchronized in their developmental staging throughout anthesis, slight deviations exist between rice and barley/wheat regulating programmed cell death of the tapetum (Browne et al. 2018). The tapetum degenerates slightly earlier in rice than in barley and wheat, which commences between the late tetrad stage and early young microspore stage (Browne et al. 2018; Lazarova 2003).

The *Btr-like* genes are likely associated with the cell wall. This is because of the closely related *Btr* genes (*Btr1* and *Btr2)* which appear to modulate the thickness of the cell wall to form an abscission zone for easy rachis disarticulation at maturity in the spike (Pourkheirandish et al. 2015). The wheat orthologue of the *Btr1* gene has been linked to the reduction in cell wall biosynthesis, which is consistent with the role of callase and callose secretion by the tapetum (Zhao et al. 2019).

Additionally, the stages of expression for *Btr* and *Btr-like* genes are overlapping where *Btr* genes express in the rachis when spikes are at the beginning of white anther stage and anthers are around 1 mm long (Pourkheirandish et al. 2015). This further suggests that neo-functionalization between *Btr* and *Btr-like* pairs is the result of spatial expression divergence.

Based on this evidence, we have deduced two working hypotheses that indicate the *Btr-like* genes play a role in pollen development. Our first hypothesis is that *Btr1-like* and *Btr2-like* gene products may be found in the tapetal cell wall membrane facing the locule where they regulate the secretion of callose (meiosis stage) and/or the production of callase (late tetrad and beyond). This is consistent with the biological effects of *TDR* and *OsPUB73*. We speculate that *Btr* and *Btr-like* genes perform a similar molecular function that controls the development of cell wall thickness but in a different tissue.

The alternative hypothesis is that the BTR1-LIKE and BTR2-LIKE proteins are present within the cell membrane of the meiocyte, where they could be involved in the construction of the primexine throughout the tetrad phase. This is more consistent with the function of *OsINP1* where the meiocyte alone is the location of cell wall abnormalities (i.e. the absence of the pollen aperture) (Zhang et al. 2020). This theory is also supported by the *Btr* genes’ predicted function in cell wall biosynthesis (Zhao et al. 2019). As the exine layer is a biological cell wall, the *Btr-like* genes may operate similarly in its construction. Both of these hypotheses require further investigation, an in situ hybridization of *Btr-like* transcripts would provide clarity and demonstrate the exact location of *Btr-like* expression within the anther.

## Conclusions and greater context

This study provides groundwork for a model of evolution of grain dispersal in barley, which can be applied to wheat, rye and other members of Triticeae that develop a brittle rachis. *Btr* genes are involved in grain dispersal via cell wall modification. The *Btr-like* genes, which are the ancestral copies of *Btr* genes, are likely involved in cell wall modification albeit in a completely different physical location. We postulate that barley grain dispersal evolved via duplication and neo-functionalization resulting from altered spatial expression patterns. The ancestral *Btr-like* genes are expressed in meiocytes and likely contribute to pollen development, while *Btr* genes are expressed in spikes internodes and are involved in grain disarticulation. We demonstrate here that genetic processes critical to the advent of barley domestication, those being the evolution of a brittle rachis, will likely be crucial to future efforts to domesticate other wild cereals within the Triticeae tribe.

## Supplementary Information

Below is the link to the electronic supplementary material.

Supplementary file1 (CSV 63596 kb)

Supplementary file2 (CSV 8951 kb)

Supplementary file3 (XLSX 36 kb)

Supplementary file4 (DOCX 33 kb)

Supplementary file5 (XLSX 17 kb)

Supplementary file6 (XLSX 51 kb)

Supplementary file7 (PDF 49 kb)

Supplementary file8 (PDF 31 kb)

## Data Availability

The transcriptome data have been downloaded from GenBank and all project IDs are listed in Table 1.
