# Supplementary material for: Grain dispersal mechanism in cereals arose from a genome duplication followed by changes in spatial expression of genes involved in pollen development
Source: Theor Appl Genet. 2022 Feb 22;135(4):1263–77. doi: 10.1007/s00122-022-04029-8 (PMC9033732; doi:10.1007/s00122-022-04029-8)
Supplement: Supplementary file 4 — Supplementary file4 (DOCX 33 kb) [file 122_2022_4029_MOESM4_ESM.docx]

Supplementary Data 4. Confirmation of observed expression patterns in EoRNA expression database. Corresponding V1 and BaRT genes were found using EoRNA BLAST search function. Not all Btr genes are found in EoRNA.

| Gene name | Gene ID V2 | Best match V1  (EoRNA) | Best match BaRT  (EoRNA) | EoRNA expression V1 | EoRNA expression BaRT | Comment |
| --- | --- | --- | --- | --- | --- | --- |
| Btr1 | 3HG0195510 | - | - |  |  |  |
| Btr1-like-a | 3HG0195460 | HORVU3HR1G018350 | BART1_0-u18463 | No TPM values available. | 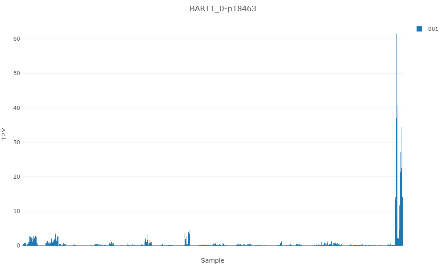 | Expression specific to anther (PRJNA558196). |
| Btr1-like-b1 | 3HG0195170 | HORVU3HR1G018140 | BART1_0-u18441 | No TPM values available. | 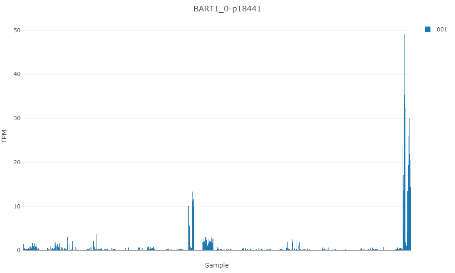 | Expression specific to microspore (PRJNA428086) and anther (PRJNA558196). |
| Btr2-like-b1 | 3HG0195160 | - | - |  |  |  |
| Btr2-like-b2 | 3HG0195470 | - | BART1_0-u18462 |  | 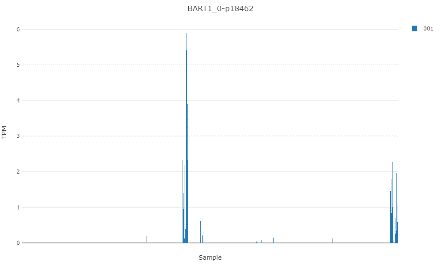 | Expression specific to microspore (PRJNA428086) and anther (PRJNA558196). |
| Btr2-like-a | 3HG0195480 | - | BART1_0-u18460 |  | 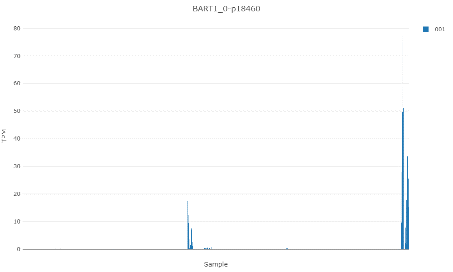 | Expression specific to microspore (PRJNA428086) and annther (PRJNA558196). |
| Btr2 | AKV61765.1 | - | - |  |  |  |
