# Supplementary material for: Grain dispersal mechanism in cereals arose from a genome duplication followed by changes in spatial expression of genes involved in pollen development
Source: Theor Appl Genet. 2022 Feb 22;135(4):1263–77. doi: 10.1007/s00122-022-04029-8 (PMC9033732; doi:10.1007/s00122-022-04029-8)
Supplement: Supplementary file 7 — Supplementary file7 (PDF 49 kb) [file 122_2022_4029_MOESM7_ESM.pdf]

TDR Multiple Sequence Alignment by MUSCLE (3.8)

```
LOC_Os02g02820.1 (TDR)           -MGRGDHLLMKNSNA-AAAAAAVNGGGTSLDAALRPLV-GSDGWDYCIYWRLSPDQRFLE
HORVU.MOREX.r2.6HG0456990.1      MGGGGDYHQQSIIGG-AAAHGHGGGGGAAVEAALRPLVGGSHGWDYCIYWRLSPDQRFLE
KAF7088250.1                     -MAGGDYHQQSIIGGRAAVHGHGGGGGGTVEAALRPLVGGAHGWDYCIYWRLSPDQRFLE
                                   . **:   .  .. **. .  .*** :::******* *: ******************

LOC_Os02g02820.1 (TDR)           MTGFCCSSELEAQVSALLDLPSSIPLDSSSIGMHAQALLSNQPIWQSSSEEE--------
HORVU.MOREX.r2.6HG0456990.1      MTGFCCSAEFEAEVAALGEIPATIPLDSSSIGMHAQALLSNQPIWQSSGGAPGPDLLTGY
KAF7088250.1                     MTGFCCSAEFEAQVATLADVPSSIPLDSSSIGMHAQALLSNQPIWQSSGGAPGPDLLTGY
                                 *******:*:**:*::* ::*::*************************.

LOC_Os02g02820.1 (TDR)           EADGGGGAKTRLLVPVAGGLVELFASRYMAEEQQMAELVMAQCGGGGAGDDGGGQAWPPP
HORVU.MOREX.r2.6HG0456990.1      EAASNGGEKTRLLVPVAGGIVELFASRYMAEEQQMAELVMAQCG-------GGGQAWQET
KAF7088250.1                     EASSSGGEKTRLLVPVAGGIVELFASRYMAEEQQMAELVMAQCG-------GGGQGWQET
                                 ** ..** ***********:************************       ****.*  .

LOC_Os02g02820.1 (TDR)           ETPSFQWD--GGADAQRLMYGGSSLNLFDAA--AADDDPFLGGGGGDAVGDEAAAAGAWP
HORVU.MOREX.r2.6HG0456990.1      EPQGFAWDAAAAADPGRL-YAAASLNLFDGAGGSGSGEPFLAG-----VQEDGGAGLGWQ
KAF7088250.1                     EAQGFAWD-AAAADPGRL-YAAASLNLFDGAGGSGSGEPFLAG-----VQEDGAAGVGWQ
                                 *. .* **  ..**. ** *..:******.*  :...:***.*     * ::..*. .*

LOC_Os02g02820.1 (TDR)           YAGMAVSEPSVAVAQEQMQ-HAAGGGVAESGSEGRKLHGGDPEDDGDGE-----GRSGGA
HORVU.MOREX.r2.6HG0456990.1      YAAESSEPPSTVQAQEYQQVHGSGVGRADSGSEGSDMQLGDPDDDGDGETQRGSGKDGGG
KAF7088250.1                     YAAESSEPPST-VAQEHQQLHGSGVGRADSGSERSDMQLGDPDDNVDGETQRGSGKDGGG
                                 **. : . **.  ***  * *.:* * *:****  .:: ***:*: ***     *..**.

LOC_Os02g02820.1 (TDR)           KRQQCKNLEAERKRRKKLNGHLYKLRSLVPNITKMDRASILGDAIDYIVGLQKQVKELQD
HORVU.MOREX.r2.6HG0456990.1      KRQQCKNLVAERRRRKKLNDRLYKLRSLVPNITKMDRASILGDAIDYIVGLQKQVKDLQD
KAF7088250.1                     KRQQCKNLIAERKRRKKLNNRLYTLRSLVPNITKMDRASILGDAIDYIVGLQKQVKDLQD
                                 ******** ***.******..**.********************************:***

LOC_Os02g02820.1 (TDR)           ELEDNHV------HHKPPDVLI-DHPPPASLVGLDNDDASPPNSHQQQPPLAVSGSSSRR
HORVU.MOREX.r2.6HG0456990.1      ELEDPNPAGGAGGDSKAPDVLLDDHPPP----GLDNDEDSP----QQQPFPSAGGKRARK
KAF7088250.1                     ELEDPNPPGVTGGHSKAPDVLLDDHPPP----GLDNDEDSP----QQQPFPSAAGKRPRK
                                 **** :         *.****: *****    *****: **    ****  :..*. .*.

LOC_Os02g02820.1 (TDR)           SNKDPAMTDDKVGGGGGGGHRMEPQLEVRQVQGNELFVQVLWEHKPGGFVRLMDAMNALG
HORVU.MOREX.r2.6HG0456990.1      EEAGDEEEKE------AEDQDMEPQVEVRQVEGKEFFLQVLCSHKSGRFVRIMDEIAALG
KAF7088250.1                     VEAGEEEEKE------AEDQDMEPQVEVRQVEGKEFFLQVLCSHKSGRFVRVMDEIAALG
                                  : .    .:      . .: ****:*****:*:*:*:*** .**.* ***:** : ***

LOC_Os02g02820.1 (TDR)           LEVINVNVTTYKTLVLNVFRVMVRDSEVAVQADRVRDSLLEVTRETYPGV--WPSPQ---
HORVU.MOREX.r2.6HG0456990.1      LQITSINVTSYNKLVLNVFRAVMKDNEAAVPADRVRDSLLEVTREMYGGGGGWSSPLPQP
KAF7088250.1                     LQITSVNVTSYNKLVLNVFRAVMKDNEAAVPADRVRDSLLEVTREMYGGGGAWSSPL--P
                                 *:: .:***:*:.*******.::.*.*.** ************** * *   *.**

LOC_Os02g02820.1 (TDR)           --EEDDAKFDGGDGGQAAAAAAAAGGEHY---HDEVGGGYHQHLHYLAFD
HORVU.MOREX.r2.6HG0456990.1      PAPPTSAKLDGMDG----QAVPAAAGDHYQLHHQVLGGYHHQHLQYLAMD
KAF7088250.1                     PPPPTNAKLDGMDG----QAVPAAAGDHYQLHHQVLGGYHHQHLQYLAMD
                                      .**:** **     *..**.*:**   *: :** :****:***:*
```

OsYABBY7 Multiple Sequence Alignment by MUSCLE (3.8)

```
LOC_Os07g38410.1 (OsYABBY7)      MSSAARHHCS-GLRERLGCVQCSFCATVLLVSVPCSSVLRVVAVQCGHCSGILSAVNLPP
HORVU.MOREX.r2.2HG0109560.1      MSSAASPLALGGLHERLGYVQCRFCATILLVSVPCSGLLKMVAVQCGRCAGILSVSVASP
KAF7015187.1                     MSSSASLPAL-GLPERLGYVQCRFCNTILLVNVPCSGLLKMVAVQCGRCAGILSVSVASP
                                 ***:*   .  ** **** *** ** *:***.****.:*.:******.*:****.   .*

LOC_Os07g38410.1 (OsYABBY7)      SP--VSASIELTPQELDAGPPPGEYSDESSGDDR--EGRDAEDDAPAPAAAAVANKPPGR
HORVU.MOREX.r2.2HG0109560.1      PPPSPPPSVELPLQELGVDPPPREWSDESSAGDNDDDDDDGEGEVVEKSATAV-NKPPVR
KAF7015187.1                     PP--PPPSVELPLQELGVDPPPREWSDDSSAGDN--DDGDWEGEVVEKSATAV-NKPPVR
                                 .*   ..*:**. ***...*** *:**:**..*.  :. * *.:.   :*:** **** *

LOC_Os07g38410.1 (OsYABBY7)      KQRTPSAYNCFVKEEIKRIKSMEPNITHKQAFSTAAKNWAHLPRIQQKRGRDSC
HORVU.MOREX.r2.2HG0109560.1      KQRTPSAYNCFIKEEIKRIKAMEPDITHKEAFSTAAKNWAHLPRIQHKEGD---
KAF7015187.1                     KQRTPSAYNCFIKEEIKRIKAMEPDITHKEAFSTAAKNWAHLPRIQHK-GD---
                                 ***********:********:***:****:****************:* *
```

OsPUB73 Multiple Sequence Alignment by MUSCLE (3.8)

```
LOC_Os02g28870.1 (OsPUB73)       ---------------MDPEAEEAQLRLEMELAKKAKADMSGLQRSSSLGLDHAGLYPLPL
KAF7060109.1                     MSGRRNRGGASAPPALAPPGPGERPPPPSQSSRRPAAATLEQRLRSSVEREIDEASAMQP
HORVU.MOREX.r2.5HG0413550.1      MPGRRNRGGASAPAGVAPPGPGERPR--SRSSRTPAAATLEQRLRRSVEREINEARAMQR
                                                : * .   .       :. . *     .   *:  :     .:

LOC_Os02g28870.1 (OsPUB73)       PPGWRSAPTSPLRTPSSPPPLQFPPAWAAD----------VAGTSGSAAPEDDGPARNAG
KAF7060109.1                     AEAGQGSSSP---APASSRPRFWPRARQAARKVLGISKKPSARSAAGTPHGQDTVPDASG
HORVU.MOREX.r2.5HG0413550.1      AEAGQGSSSPSPSAPASSRSRFWPRARQAARKVLGISKKPSPGSAAGTPHGQETVPEAAG
                                 . . ..:.:.   :*:*. .  :* *. *            . ::..:.  ::  .  :*

LOC_Os02g28870.1 (OsPUB73)       ADE----ATAGSAPKNEDPARAAGADDGPTRSDYAAMMRMALAKFQDDDAAA--------
KAF7060109.1                     TSESAAVAAARTGTGDEAGSEQQPVEVAPTRSEFAAMMQTALAKIQEGDAADDQAKRQAA
HORVU.MOREX.r2.5HG0413550.1      TSE----SAARTGPGDEARSEQQPVVVAPARSEFAAMMQSALAKIQEGATAD--------
                                 :.*    ::* :.. :*  :    .  .*:**::****. ****:*:. :*

LOC_Os02g28870.1 (OsPUB73)       -------------------------------DDEEAASAVMEQAMTGLMDLTYRKAK-P
KAF7060109.1                     IAAMEKAMTFAEMEKAMTALMKIQEDAAVGDQAQGQAAFAEMEKAMTGLMDLSHKKTSGP
HORVU.MOREX.r2.5HG0413550.1      -----------------------------DQAQGQAAFAEMEKAMTGLMDLSHKKTSGP
                                                                  : :** * **:********::.*:. *

LOC_Os02g28870.1 (OsPUB73)       PELPYEFATRWPIPIAHDGTLQAEVMRDPVILPSGYSVDQTYQNNQKRQNPWTNTSTFTD
KAF7060109.1                     PKLPRDFATKW--PHSEGDPLLERVMKDPIILASGYTVDKSCQQWSLAQK---NTCPVTG
HORVU.MOREX.r2.5HG0413550.1      PKLPRDFATRW--PHSDGDPLLERVMKDPIILASGHTVDKSCQQWSVAHK---NTCPVTG
                                 *:** :***.*  * : ...*   **.**:**.**::**:: *:..  ::   **...*.

LOC_Os02g28870.1 (OsPUB73)       HSLPYSLSVPNHLLRDMISAWCLDHSDLSPSTTSDTPSTPLEPSEEEQIQRILKLFSGNS
KAF7060109.1                     HSLPHSLTAPNHLLHDMIAEWCLDHSNLARSSIG--RSLPLVPPAEDEIQEILELFSGHP
HORVU.MOREX.r2.5HG0413550.1      HSLPHSVTAPNHLLHDMIAEWCLDHSNLARSSTGVTRSLPLVPPSEDQIQDILELFSGHS
                                 ****:*::.*****.***: ******:*: *: .   * ** *. *::** **:****:.

LOC_Os02g28870.1 (OsPUB73)       ASQREALKLIQLLTKTTKGVQPCLAKYADIIPVLINLRRKYKSSWTQDLEEERLTIILNL
KAF7060109.1                     VRQKEALRMLNLMSKTSKGMQPCLAKWPELTPLLMNLRKHWMNVWSADIEAQRISLIHNL
HORVU.MOREX.r2.5HG0413550.1      VRQKEALRLINLMSKTSKGMKPCLDKWPELIPLLIDLRKEWNNVWSADIEAQRISLFHNL
                                 . *.***.:::*::**:**::*** *:.:: *:*::**. : . *: *:* :*:::: **

LOC_Os02g28870.1 (OsPUB73)       TMHRQNREILAGQNELAGAIKKIVKKAGNRGKRTSSLAKVASIVAVLSEFDMFRKRMLDA
KAF7060109.1                     SMHRPNREILAGQNEVPAVLKNVVERAGKLGLSASLLAMVASIIATLSEFDVFRKRMVTI
HORVU.MOREX.r2.5HG0413550.1      SIHRPNREILACQNELPAVLKNVVERAGRLGISASVLAMVASVVATLSEFDVFRKRMVMI
                                 ::** ****** ***:...:*::*:.**. *  :* ** ***::*.*****:*****:

LOC_Os02g28870.1 (OsPUB73)       GGMKMLRGMLKIKDTEVITEAATAILALYADGEGEQPARFHEVPQMLLECHMFTDGILLL
KAF7060109.1                     GGMKMLSGLLKIEDVVLRKETGAAILALCADEEAKLSAAVSDVPDRLLECFMATDEFLLL
HORVU.MOREX.r2.5HG0413550.1      GGMKMLTGLLKIEDVVVRKETGAAILALCADKEAKLSAAVNDVPDKLLGCFMATDEFLLL
                                 ****** *:***:*. : .*:.:***** ** *.: .* . :**: ** *.* ** :***

LOC_Os02g28870.1 (OsPUB73)       LDRLPKSPRVFRKICDQALQLVNIVMAEDASGPVTRKGILSAISLIYEIVERDVGKMNAV
KAF7060109.1                     LDRLPKSPEALDMICDKAMELVNIVIEEDAGGMVTSQGIHSAISLIFVITERDVGKLK-V
HORVU.MOREX.r2.5HG0413550.1      LHRLPKSPEALDMICDKAVELVNIVMGDDAGGMVTSQGIHSAISLIFVITERDVGKLK-V
                                 * ****** .:  ***:*::*****: :**.* ** :** ******: *.******:: *

LOC_Os02g28870.1 (OsPUB73)       KNMEDFIERLRQLSSDRLPMQKMLQVERIIRTLSDAFPAPTVRGRCQEPSGSRLLA
KAF7060109.1                     KNVEDFKERLRELSSKRIPMQTMFQVEKIIKTLSEMFPAPTTQLQNQ---------
HORVU.MOREX.r2.5HG0413550.1      KNVEDFKERLRELSSKRIPMQTMFHVEEIIKTLSEMFPAPTTQLQNQ---------
                                 **:*** ****:***.*:***.*::** **.***: *****.. . *
```

OsINP1 Multiple Sequence Alignment by MUSCLE (3.8)

```
LOC_Os02g44250.1 (OsINP1)       MPRPPPPPPPGRGAPGARRPMREFFAAWLSTLRSPLLPLLRRALSSSSSSSSGGWDDPLS
HORVU.MOREX.r2.6HG0498590.1     --------------------MRDFFAAWLATLRSPLLPLLRRAL----SSSPGSWNDPIS
KAF7079703.1                    ---MPRPPPPGRGAPGARRPMRDFFAAWLATLRSPLLPLLRRAL----SSSPGAWNDPLS
                                                    **:******:**************    ***.*.*:**:*

LOC_Os02g44250.1 (OsINP1)       SAAAAVEAHFQAHWSALDAAARQDPAQAVSAGDWRSPLELPFLWVGDLHPSLVTSLLRSL
HORVU.MOREX.r2.6HG0498590.1     SAAAAVEAHFEAHWSALDSAARQDPAQVICAGDWRSPLELPFLWLGDFHPSLLTSLLRSL
KAF7079703.1                    SAAAAVDAHFEAHWSALDSAARQDPAQVICAGDWRSPLELPFLWLGDFHPSLLTSLLRSL
                                ******:***:*******:********.:.**************:**:****:*******

LOC_Os02g44250.1 (OsINP1)       SPSPRLLAATDRVDRRIRAAVPSISDRLRRVQEAFISAEVSGAADVEAFLEELKDVALDA
HORVU.MOREX.r2.6HG0498590.1     SPSPRLLASADRVDRRIRAAVPVVTDRLRHAQEVFTSAEVAGGADLEAFLEELKDVALEA
KAF7079703.1                    SPSPRLLASADRVDRRIRAAVPVLSDRLRHAQEVFTSAEVAGGADLEAFLEELKAVALEA
                                ********::************ ::****..**.* ****:*.**:******** ***:*

LOC_Os02g44250.1 (OsINP1)       NRLRRGVLSELVAAAGGYQAALFLEALSRFVLSMHDPEVLRRFDQCRASPGS-------
HORVU.MOREX.r2.6HG0498590.1     NRLRRGVLSELVAAAGGHQAALFLEALSRFVLSMHDPEVLRRFDHCRPSPGIFGRHETR
KAF7079703.1                    NRLRRGVLSDLVAAAGGHQAALFLEALSRFVLSMHDPEVLRRFDHCRPSPGIFGRHEAR
                                *********:*******:**************************:**.***
```

ITPK5 Multiple Sequence Alignment by MUSCLE (3.8)

```
LOC_Os10g42550.1 (ITPK5)        ----MAGDEPLPGDGQRRRYLIGYALAPKKQQSFIQPSLVSRAAGRGMDLVPVDPSRPLP
HORVU.MOREX.r2.1HG0041780.1     -MAAAAAAAAAEASSADSRRYVVGYALAPKKQNSFIKPSLISRAAARGVDLVPVDDARPLA
KAF6983924.1                    MAAAAAAAAAAEPSSADSRRYVVGYALAPKKQNSFIKPSLISRAAARGVDLVPVDDARPLA
                                     *.  . ....: ***::*********:***:***:****.**:****** :***.

LOC_Os10g42550.1 (ITPK5)        EQGPFHLLIHKLYGEEWRGQLDAFSAAHPAVPVVDPPHAIDRLHNRISMLQVVSELDVPL
HORVU.MOREX.r2.1HG0041780.1     DQGPFDLVIHKLYGHDWRAQLQAFSARYPSVPVVDPPHAIDRLHNRISMLQVVSELDVPP
KAF6983924.1                    DQGPFDLVIHKLYGHDWRAQLQAFSARYPSVPVVDPPHAIDRLHNRISMLQVVSELDVPL
                                :**** *:****** :**.**:**** :*:*****************************

LOC_Os10g42550.1 (ITPK5)        HA---------HHHHTFGIPSQVVVYDAAALSDSGLLAALRFPLIAKPLVADGTAKSHKM
HORVU.MOREX.r2.1HG0041780.1     GDA--------DRRDTFGIPSQVVVYDAAALADSGLLAALRFPLIAKPLVADGSAKSHKM
KAF6983924.1                    TDAASASADGEGHRHTFGIPSQVVVYDAAALADSGLLAALRFPLIAKPLVADGSAKSHKM
                                            .. ****************:*********************:******

LOC_Os10g42550.1 (ITPK5)        SLVYHREGLRKLRPPLVLQEFVNHGGVIFKVYVVGAHVTCVKRRSLPDVSSDVLQDASAE
HORVU.MOREX.r2.1HG0041780.1     SLVYHREGLRKLRPPLVLQEFVNHGGVIFKVYVVGGHVTCVKRRSLPDVSKEILEDAAAE
KAF6983924.1                    SLVYHREGLRKLRPPLVLQEFVNHGGVIFKVYVVGGHVTCVKRRSLPDVSKEILEDAAAE
                                ***********************************.**************.::*:**:**

LOC_Os10g42550.1 (ITPK5)        GSLSFSQVSNLPNERTAQEYYDDMRLEDAIMPPTAFINDIAAALRRALGLHLFNFDMIRD
HORVU.MOREX.r2.1HG0041780.1     GTISFSQVSNLPNQRTAEEYYEDMRLEDAVMPPTDFVNEIAGGLRRALGLQLFNFDMIRD
KAF6983924.1                    GTISFSQVSNLPNQRTAEEYYEDMRLEDAVMPPTDFVNEIAGGLRRALGLQLFNFDMIRD
                                *::**********:***:***:*******:**** *:*:**..*******:*********

LOC_Os10g42550.1 (ITPK5)        ARAGDRYLVIDINYFPGYAKMPGYETVLTDFFWEMVHKDD-DTPNLNPNPNDEDVK
HORVU.MOREX.r2.1HG0041780.1     VRAGDRYLVIDINYFPGYAKMPGYEIVLTDFFWDMVHKDDVALKEEEEQSNHAVVK
KAF6983924.1                    VRAGNRYLVIDINYFPGYAKMPGYEIVLTDFFWDMVHKDDVALKEEEQQSNHAVVK
                                .***:******************** *******:******    : : :.*   **
```

OsAsp1 Multiple Sequence Alignment by MUSCLE (3.8)

```
LOC_Os11g08200.1 (OsAsp1)        -------MTARLAL-LASLLLLLQLVPPSSAVVLELHGNVYPIGHFFITMNIGDPAKSYF
HORVU.MOREX.r2.4HG0289080.1      -------MAAIWTMVIVHLLLLLPLL--RSSIVFELHGDVYPTGLFYVTMNIGEPAKPYN
KAF7052952.1                     MSSSSTTMAAIWALAIIHLLLLLPLL--RSSIVFELHGDVYPTGLFYVTMNIGEPAKPYN
                                        *:*  :: :  ***** *:   *::*:****:*** * *::*****:***.*

LOC_Os11g08200.1 (OsAsp1)        LDIDTGSTLTWLQCDAPCTNCNIVPHVLYKPTPKKLVTCADSLCTDLYTDLGKPKRCG-S
HORVU.MOREX.r2.4HG0289080.1      LDVDTGSPLTWLECNAPLKSTHKGPHEPYRPTRTNLVPCEDERCVAVHRDLGLAHDCTQN
KAF7052952.1                     LDVDTGSPLTWLECDAPLQSTHKGPHEAYRPTPTNVVPCDDERCVAVHRDLGLAHDCTRN
                                 **:****.****:*:**  . :  **  *.** .::*.* *. *. :: *** .: *  .

LOC_Os11g08200.1 (OsAsp1)        QKQCDYVIQYVDS-SSMGVLVIDRFSLSASNGTNPTTIAFGCGYDQ--GKKNRNVPIPVD
HORVU.MOREX.r2.4HG0289080.1      PHQCDYVLRYKDGESSLGVLLIDQFSLTTNNENRP-YLAFGCGYDQEGGQEARAGPVEVD
KAF7052952.1                     PDQCDYVFGYKDGESSLGVLLADQFSLPTNNENRP-NLAFGCGYDQEGGQEAGKKLVEAD
                                   *****: * *. **:***: *.***.:.* ..*  :********  *::     : .*

LOC_Os11g08200.1 (OsAsp1)        SILGLSRGKVTLLSQLKSQGVITKHVLGHCISSKGGGFLFFGDAQVPTSGVTWTPMNREH
HORVU.MOREX.r2.4HG0289080.1      GVLGIGRGTGDLVSQLRQQGIITQHIFGHCLGVRGGGFLFFGGDRVPSAGVTWVPMAQNV
KAF7052952.1                     GVLGIGRGTGDLVSQLKQQGIITDNIFGHCLGVHGGGFLFFGGDRVPSAGVTWVPMAQNV
                                 .:**:.**.  *:***..**:**.:::***:. .********. .**::****.** .:

LOC_Os11g08200.1 (OsAsp1)        KY-YSPGHGTLHFDSNSK-AISAAPMAVIFDSGATYTYFAAQPYQATLSVVKSTLNSECK
HORVU.MOREX.r2.4HG0289080.1      RSHYSPGAATLNLNVQLEYPVSVEPMPTIFDSGSTYTYVHMDTYARLISAVAVTLESSS-
KAF7052952.1                     GSHYSPGAATLNLNVQLEYPVSMEPMTTMFDSGSTYTYVHKDTYGRLISAVGVTLEGSS-
                                    **** .**::: : : .:*  **..:****:****.  :.*   :*.*  **:...

LOC_Os11g08200.1 (OsAsp1)        FLTEVTEKDRALTVCWKGKDKIVTIDEVKKCFRSLSLEFADGDKKATLEIPPEHYLIISQ
HORVU.MOREX.r2.4HG0289080.1      -LTKA--HDDALPECWEENELIQSVDNVKNKFKPLEFTFGHGVNQATMEIPPENYIVVTA
KAF7052952.1                     -LTKV--DDDALAECWEENEPIQFVDDVKSKFKPLELTFGHGANQATMEIPPENYIVVTK
                                  **:.   * **. **: :: *  :*:**. *..*.: *. * ::**:*****:*::::

LOC_Os11g08200.1 (OsAsp1)        EGHVCLGILDGSKEHLSLAGTNLIGGITMLDQMVIYDSERSLLGWVNYQCDRIPRSESAI
HORVU.MOREX.r2.4HG0289080.1      SKP-----------------------------------------WVDH------------
KAF7052952.1                     TGKVCLGILNGSQ--IGLDRLNLIGGNTMQNYIMIYDNERARIGWARASCYEMPGLEPLI
                                                                             *.

LOC_Os11g08200.1 (OsAsp1)        TSRL
HORVU.MOREX.r2.4HG0289080.1      ----
KAF7052952.1                     GSRL
```
