# Supplementary material for: Grain dispersal mechanism in cereals arose from a genome duplication followed by changes in spatial expression of genes involved in pollen development
Source: Theor Appl Genet. 2022 Feb 22;135(4):1263–77. doi: 10.1007/s00122-022-04029-8 (PMC9033732; doi:10.1007/s00122-022-04029-8)
Supplement: Supplementary file 8 — Supplementary file8 (PDF 31 kb) [file 122_2022_4029_MOESM8_ESM.pdf]

OsAsp1 Homologues Multiple Sequence Alignment by MUSCLE (3.8)

```
HORVU.MOREX.r2.4HG0289080.1 (nucellin-like)     -MAAIW--TMVIVHLLLLLPLLR---------------SSIVFELHGDVYPTGLFYVTMN
LOC_Os11g08200.1 (OsAsp1)                       -MTARL---ALLASLLLLLQLVP-------------PSSAVVLELHGNVYPIGHFFITMN
KAE8783989.1 (nucellin)                         MAPARWSPTAFLAVLLLLPPFAPSPARAATPGKSLSSASTAVFQLQGAVYPIGHYYVTMN
                                                  .*      .:. ****  :                 *: *::*:* *** * :::***

HORVU.MOREX.r2.4HG0289080.1 (nucellin-like)     IGEPAKPYNLDVDTGSPLTWLECNAPLKSTHKGPHEPYRPTRTNLVPCEDERCVAVHRDL
LOC_Os11g08200.1 (OsAsp1)                       IGDPAKSYFLDIDTGSTLTWLQCDAPCTNCNIVPHVLYKPTPKKLVTCADSLCTDLYTDL
KAE8783989.1 (nucellin)                         IGDPAKPYFLDVDTGSDLTWLQCDAPCQSCNKVPHPWYKPTKNKIVPCAASLCTSLTPN-
                                                **:***.* **:**** ****:*:**  . :  **  *.** .::*.*  . *. :  :

HORVU.MOREX.r2.4HG0289080.1 (nucellin-like)     GLAHDCTQNPHQCDYVLRYKDGESSLGVLLIDQFSLTTNNEN--RPYLAFGCGYDQEGGQ
LOC_Os11g08200.1 (OsAsp1)                       GKPKRCG-SQKQCDYVIQYVD-SSSMGVLVIDRFSLSASNGTN-PTTIAFGCGYDQ--GK
KAE8783989.1 (nucellin)                         ---KKCA-VPQQCDYQIKYTDKASSLGVLIADNFTLSLRNSSTVRANLTFGCGYDQQVGK
                                                   : *    :**** :.* *  **:***: *.*:*:  * .   . ::*******  *:

HORVU.MOREX.r2.4HG0289080.1 (nucellin-like)     EARAGPVEVDGVLGIGRGTGDLVSQLRQQGIITQHIFGHCLGVRGGGFLFFGGDRVPSAG
LOC_Os11g08200.1 (OsAsp1)                       KNRNVPIPVDSILGLSRGKVTLLSQLKSQGVITKHVLGHCISSKGGGFLFFGDAQVPTSG
KAE8783989.1 (nucellin)                         -NGAVQAATDGLLGLGKGAVSLLSQLKQQGV-TKNVLGHCFSTNGGGFLFFGDDIVPTSR
                                                        .*.:**:..*   *:***..**: *::::***:. .********.  **::

HORVU.MOREX.r2.4HG0289080.1 (nucellin-like)     VTWVPMAQNVR-SHYSPGAATLNLNVQLEYPVSVEPMPTIFDSGSTYTYVHMDTYARLIS
LOC_Os11g08200.1 (OsAsp1)                       VTWTPMNREHK--YYSPGHGTLHFDSNSK-AISAAPMAVIFDSGATYTYFAAQPYQATLS
KAE8783989.1 (nucellin)                         VTWVPMARTTSGNYYSPGSGTLYFDRR---SLGMKPMEVVFDSGSTYAYFAAEPYQATVS
                                                ***.** .     :**** .** :: .   .:.  ** .:****:**:*.  :.*   :*

HORVU.MOREX.r2.4HG0289080.1 (nucellin-like)     AVAVTLESSS--LTKAH--DDALPECWEENELIQSVDNVKNKFKPLEFTFGHGVNQATME
LOC_Os11g08200.1 (OsAsp1)                       VVKSTLNSECKFLTEVTEKDRALTVCWKGKDKIVTIDEVKKCFRSLSLEFADGDKKATLE
KAE8783989.1 (nucellin)                         ALKAGLSKS---LKEVS--DVSLPLCWKGQKVFKSVSEVKNDFKSLFLSFG---KNSVME
                                                .:   *...   *.:.   * :*. **: :. : ::.:**: *..* : *.   :::.:*

HORVU.MOREX.r2.4HG0289080.1 (nucellin-like)     IPPENYIVVTASKP-----------------------------------------WVDH-
LOC_Os11g08200.1 (OsAsp1)                       IPPEHYLIISQEGHVCLGILDGSKEHLSLAGTNLIGGITMLDQMVIYDSERSLLGWVNYQ
KAE8783989.1 (nucellin)                         IPPENYLIVTKYGNVCLGILDGTTAKLKF---NIIGDITMQDQMIIYDNEKGQLGWIRGS
                                                ****:*::::                                             *:

HORVU.MOREX.r2.4HG0289080.1 (nucellin-like)     ---------------
LOC_Os11g08200.1 (OsAsp1)                       CDRIPRSESAITSRL
KAE8783989.1 (nucellin)                         CSR--SSKSLMSSFP
```

OsAsp1 Homologues Percent Identity Matrix - created by Clustal2.1

1: HORVU.MOREX.r2.4HG0289080.1 (nucellin-like)	100.00	48.27	48.97
2: LOC_Os11g08200.1 (OsAsp1)	48.27	100.00	57.84
3: KAE8783989.1 (nucellin)	48.97	57.84	100.00
